# Pyroptosis in the Retinal Neurovascular Unit: New Insights Into Diabetic Retinopathy

**DOI:** 10.3389/fimmu.2021.763092

**Published:** 2021-10-19

**Authors:** Chunren Meng, Chufeng Gu, Shuai He, Tong Su, Thashi Lhamo, Deji Draga, Qinghua Qiu

**Affiliations:** ^1^ Department of Ophthalmology, Shanghai General Hospital, Shanghai Jiao Tong University School of Medicine, Shanghai, China; ^2^ National Clinical Research Center for Eye Diseases; Shanghai Key Laboratory of Ocular Fundus Diseases, Shanghai Engineering Center for Visual Science and Photomedicine, Shanghai Engineering Center for Precise Diagnosis and Treatment of Eye Diseases, Shanghai, China; ^3^ Department of Ophthalmology, Shigatse People’s Hospital, Shigatse, China

**Keywords:** diabetic retinopathy, retinal neurovascular unit, pyroptosis, inflammation, IL-1β and IL-18

## Abstract

Diabetic retinopathy (DR) is prevalent among people with long-term diabetes mellitus (DM) and remains the leading cause of visual impairment in working-aged people. DR is related to chronic low-level inflammatory reactions. Pyroptosis is an emerging type of inflammatory cell death mediated by gasdermin D (GSDMD), NOD-like receptors and inflammatory caspases that promote interleukin-1β (IL-1β) and IL-18 release. In addition, the retinal neurovascular unit (NVU) is the functional basis of the retina. Recent studies have shown that pyroptosis may participate in the destruction of retinal NVU cells in simulated hyperglycemic DR environments. In this review, we will clarify the importance of pyroptosis in the retinal NVU during the development of DR.

## 1 Introduction

Diabetes mellitus (DM) is a prevalent metabolic disorder syndrome causing multiple systemic complications (1). According to the International Diabetes Federation, the global incidence of DM will increase in the next few decades, from an estimated 9.3% in 2019 to 10.2% by 2030 and 10.9% by 2045 (2). DR is a common and progressive microvascular complication of DM that can cause irreversible retinal damage (3) and it remains the main cause of impaired vision in working-aged people (4). According to a meta-analysis, the global population with moderate or more severe vision impairment due to DR was 2.6 million in 2015, and the number is predicted to increase to 3.2 million by 2020 (5). Thus, DR will impose a heavy economic burden on individuals and society worldwide. DR is mainly caused by hyperglycemia. Long-term hyperglycemia can cause characteristic pathological changes in the retina, such as thickening of the basement membrane of the retinal microvessels, loss of vascular cells, increased vascular permeability, and neovascularization (6). A better understanding of the pathogenesis in the retina is urgently needed to develop interventions. According to the International Council of Ophthalmology, DR falls into two categories: nonproliferative DR (NPDR) and proliferative DR (PDR, **Table 1**) (7). NPDR is regarded as the early stage of DR and PDR is the advanced stage. When DR affects the macula, it can cause diabetic macular edema (DME). DME can occur in any stage of DR and is the most frequent cause of blindness in diabetic patients (8).

**Table 1:** Classification of Diabetic Retinopathy.

Classification	Defining changes
Normal retina	No abnormality
Mild NPDR	Only microaneurysms
Moderate NPDR	Microaneurysms and one or more of following findings: Dot and blot hemorrhages Hard exudation Cotton wool spots
Severe NPDR	Any one of these findings: ≥ 20 intraretinal hemorrhages in each quadrant Beaded veins in two quadrants IRMAs in one quadrant
PDR	One or more of these changes: Neovascularization Preretinal hemorrhages Vitreous hemorrhage

DR, diabetic retinopathy; NPDR, non-proliferative DR; IRMAs, intra-retinal microvascular anomalies; PDR, proliferative DR.

**Figure 1:**
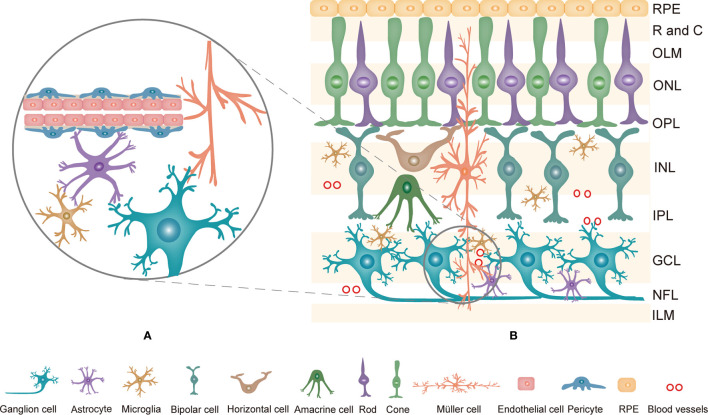
**(A)** The composition of the retinal NVU. Neurons, Müller cells, astrocytes, microglia, endothelial cells and pericytes are linked together to form the retinal NVU. **(B)** Structure of the retina and the distribution of retinal NVU components in the retina. The retina is generally divided into ten layers. Retinal neurons, various glial cells and blood cells are distributed in the corresponding layers in the retina. NVU, neurovascular unit; RPE, Retinal pigment epithelial; R and C, rod and cone; OLM, outer limited membrane; ONL, outer nuclear layer; OPL, outer plexiform layer; INL, internal nuclear layer; IPL, internal plexiform layer; GCL, ganglion cell layer; NFL, nerve fiber layer; ILM, internal limited membrane.

The retina is a complex system consisting of the retinal pigment epithelium (RPE) and the neurosensory retina. Generally, the retina is comprised of ten layers, from the outside to the inside: RPE, rod and cone layer, outer limited membrane (OLM), outer nuclear layer (ONL), outer plexiform layer (OPL), internal nuclear layer (INL), internal plexiform layer (IPL), ganglion cell layer (GCL), nerve fiber layer (NFL) and internal limited membrane (ILM) (9). Histologically, neurons, glia, and blood cells in the retina are linked together to form an important structure named the retinal neurovascular unit (NVU, **Figure 1A**) (10). The NVU consists of retinal neurons (photoreceptors: cones and rods, horizontal and bipolar cells, amacrine cells, and ganglion cells), glial cells (Müller cells, astrocytes, and microglia) and blood cells (endothelial cells and pericytes) (11). All components of the retinal NVU have different distributions in the ten-layer structure of the retina (**Figure 1B**) (9). Accumulating studies have suggested that these factors are related to the pathogenesis of DR, namely, retinal microangiopathy, retinal neurodegeneration and inflammation (6). In recent years, chronic inflammation has been shown to be the key to pathological changes in the NVU (12, 13). Pyroptosis is a novel inflammatory form of regulated cell death that facilitates the release of many proinflammatory factors, including interleukin-1β (IL-1β) and IL-18 (14, 15). Multiple studies have revealed that pyroptosis is relevant to the development of DR. This review describes the currently available studies examining the effect of pyroptosis in the retinal NVU on DR. We will focus on the retinal NVU, pyroptosis, and the effect of the latter on the former.

## 2 Retinal NVU in DR

The NVU is an important structure of the retina. As mentioned above, the retinal NVU mainly includes six components: retinal neurons, endothelial cells and pericytes, Müller cells, astrocytes and microglia. Each component of the retinal NVU has different physiological functions, but a close relationship exists among them. As more in-depth research on DR has been conducted, it has come to be regarded as a NVU disease (16). During the development of DR, many mechanisms lead to pathological changes in the retina, including oxidative stress, endoplasmic reticulum stress and inflammation (17–20).

### 2.1 The Retinal NVU

The retinal microvascular system is an indispensable constituent of the normal retina (21). The distribution of the microvasculature in the retina is specific, not spread through all layers of the retina. Currently, the microvasculature is found in four layers of the retina: (1) the deep part of the INL, (2) the border of the shallow INL and deep IPL, (3) the shallow part of the IPL and the RGCL, and (4) the NFL, and the photoreceptor layer is devoid of retinal blood flow (22, 23). Vascular endothelial cells and pericytes are important components of the retinal microvasculature, sharing a common basement membrane (24). The structure and function of the microvasculature rely on interactions between pericytes and endothelial cells, which are disturbed in some retinal vascular diseases, such as DR, retinal vascular occlusion and retinopathy of prematurity (21). Additionally, pericytes and endothelial cells are important components of the retinal NVU, and interactions between the two types of cells are necessary for the complete structure and normal function of the retinal NVU (21). In addition, the pericyte-endothelial interaction is an essential component of the internal blood retina-barrier (BRB), which is a highly selective barrier protecting the retina from the blood circulation (25, 26).

Retinal neurons are sensitive cells of various types in the retinal NVU. Retinal neurons include five major cell types: photoreceptors, horizontal cells, bipolar cells, amacrine cells and ganglion cells (27). Photoreceptors are light-sensing cells that are categorized into cone and rod cell types (28). The ONL contains the cone and rod photoreceptor cell bodies, the INL contains the cell bodies of amacrine, bipolar and horizontal cells, and the GCL mainly consists of ganglion cell bodies (29). Photoreceptor cells are in contact with secondary neurons (bipolar and horizontal cells), which in turn are in contact with ganglion cells in the IPL. In the retina, ganglion cells are the output neurons integrating information (30). Axons of ganglion cells comprise the NFL that sends the visual signal to the visual cortex through the optic nerve (31, 32).

Müller cells and astrocytes are two types of macroglia in the retina (33). Müller cells are the most abundant, accounting for 90% (34). In addition to their larger number, the distribution of Müller cells is also wide. Müller cells penetrate almost all layers of the retina and contact a variety of retinal cells (35). Due to their unique position, the normal function of Müller cells is necessary to maintain retinal homeostasis. Müller cells participate in structural support and metabolic nutrition in a healthy retina. For instance, Müller cells participate in the regulation of nutrition metabolism and protection of neurons (35). In comparison, retinal astrocytes are found only in the NFL and GCL (36). Astrocytes play a pivotal role in the metabolism and mechanical support of the neurons and serve as an essential component in the internal BRB (34, 36, 37).

In the brain and retina, microglia are resident immune cells that monitor their surroundings (38). Under normal circumstances, microglia spread over the NFL, GCL, IPL, INL, and OPL of the retina (34). According to recent research, microglia are present in the ONL only under pathological conditions (38). As immune cells of the central nervous system (CNS) (39), the functions of microglia are subdivided into six major categories: (1) Phagocytosis: Microglia predominantly clear cellular waste from the retina (40). (2) Immune Functions: Microglia are thought to participate in antigen presentation, inflammatory reactions and complement activation during defense against infectious substances and to facilitate tissue repair and immune regulation in the retina (38). (3) Microglia participate in regulating progenitor cell proliferation, differentiation, and neuronal survival (41). (4) Microglia are necessary to maintain synaptic transmission based on the synaptic structure and normal visual function in the adult retina (42). (5) Microglia have an essential role in angiogenesis. (6) Microglia are necessary to maintain retinal homeostasis (43).

**Figure 2:**
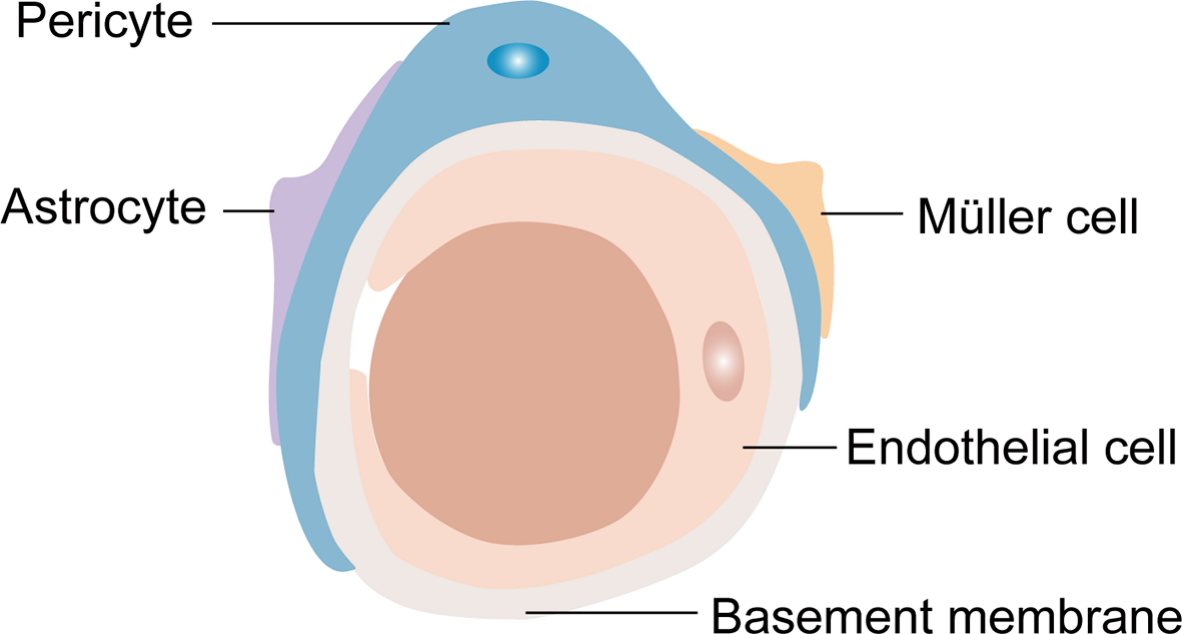
The composition of the internal blood retina barrier (iBRB). Pericytes, endfeet of astrocytes and Müller cells cover the endothelial cells to form the iBRB.

Generally, the retinal NVU participates in retinal nutrition and metabolism and provides an appropriate environment for neural signal transmission (12, 44). Furthermore, RPE cells and the retinal NVU are the core components of the BRB, an important protective barrier that is comprised of two parts: the internal BRB and the outer BRB. Most of the components of the retinal NVU are involved in the composition of the internal BRB (**Figure 2**) (45). The outer BRB is mainly comprised of tight junctions of RPE cells (46). Functionally, the internal BRB is essential in maintaining the microenvironmental homeostasis of the inner retina layers, and the outer BRB mainly regulates the transfer of solutes and nutrients from the blood to the photoreceptors (47–49).

### 2.2 Retinal NVU Changes in DR

According to previous studies, many components of the retinal NVU are affected in individuals with diabetes (50). The proper function of every element of this retinal NVU is critical for normal retinal function. In individuals with DR, damage to various cells in the retinal NVU leads to dysfunction of every component associated with the development of DR (51). The diabetic environment damages the retinal NVU through various pathways, such as oxidative stress, endoplasmic reticulum stress and inflammation (52).

#### 2.2.1 Microangiopathy in DR

In the early stage of DR, a variety of pathological changes occur in the microvascular system, such as basement membrane thickening and the loss of pericytes and endothelial cells, resulting in the destruction of the BRB and the formation of microaneurysms (53). The collected evidence indicates that retinal microvascular pathology is related to oxidative stress, apoptosis, inflammation and endoplasmic reticulum stress (54, 55). Mitochondria are the main sites for reactive oxygen species (ROS) production. High glucose (HG) increases mitochondrial production of ROS, and excessively produced ROS leads to mitochondrial dysfunction. Mitochondrial dysfunction induces apoptosis of retinal vascular cells (55). Furthermore, hyperglycemia initiates the caspase-3 activation pathway mediated by mitochondrial cytochrome C to induce retinal capillary cell death (56). Moreover, elevated ROS levels promote the expression of proinflammatory cytokines by the ROS/nuclear factor-κB (NF-κB) pathway. These proinflammatory mediators promote BRB disruption leading to microaneurysms and retinal leakage (57). The endoplasmic reticulum (ER) is mainly responsible for protein synthesis and folding in cells. Multiple studies have shown that ER stress is involved in pericyte changes in DR. Intermittent hyperglycemia promotes pericytes to secrete more macrophage chemotactic protein 1 (MCP-1), activated transcription factor 4 (ATF4) and C/EBP homologous protein (CHOP). MCP-1, ATF4 and CHOP are mediators of ER stress related to inflammation and cell death (58). Oxidative stress and ER stress promote the release of proinflammatory mediators. The inflammatory response of the retinal microvascular system is triggered by various factors, such as HG, cytokines and chemokines, and ROS, and plays a crucial role in early DR (59). Endothelial cells are extremely sensitive to proinflammatory factors. Upregulated proinflammatory factors not only induce changes in inflammatory pathways and apoptosis in endothelial cells but also stimulate endothelial cells to produce intracellular adhesion molecules, causing leukocyte stagnation (20).

#### 2.2.2 Neurodegeneration in DR

Neurodegeneration is also an important pathological change in DR that may occur before visible microvascular pathologies (60). Neuronal apoptosis is an important characteristic of neuronal degeneration. A previous study revealed an association between increased levels of protein kinase RNA-like ER kinase (PERK) and CHOP in retinal neurons of diabetic rats with retinal ganglion cell (RGC) apoptosis, similar to the results obtained from nondiabetic rats exposed to HG (61). CHOP promotes protein synthesis in the ER to cause oxidative stress and cell death (62). In addition, the hyperglycemic environment cause oxidative stress. The HG-induced increase in ROS levels promotes the apoptosis of RGCs (63). Inflammation also contributes substantially to neuronal apoptosis. For example, NF-κB activation induced by hyperglycemia is associated with RGC death in DR (64).

As mentioned above, accumulating evidence has suggested that inflammation is associated with the development of DR (65), and sustained inflammation can lead to retinal NVU component injuries (66). Pyroptosis is an emerging type of inflammatory cell death inextricably linked with inflammation. The caspase-1 mediated pathway, which is activated by NLR family pyrin domain containing 3 (NLRP3) and NLRP1 inflammasomes, is the canonical inflammasome pathway that triggers pyroptosis. Recent studies have indicated that these inflammasomes are associated with neurovascular diseases, especially those occurring in the CNS, such as DR, neurodegeneration disease and stroke (65). In other words, pyroptosis may be related to retinal NVU dysfunction under diabetic conditions.

## 3 Pyroptosis

**Figure 3:**
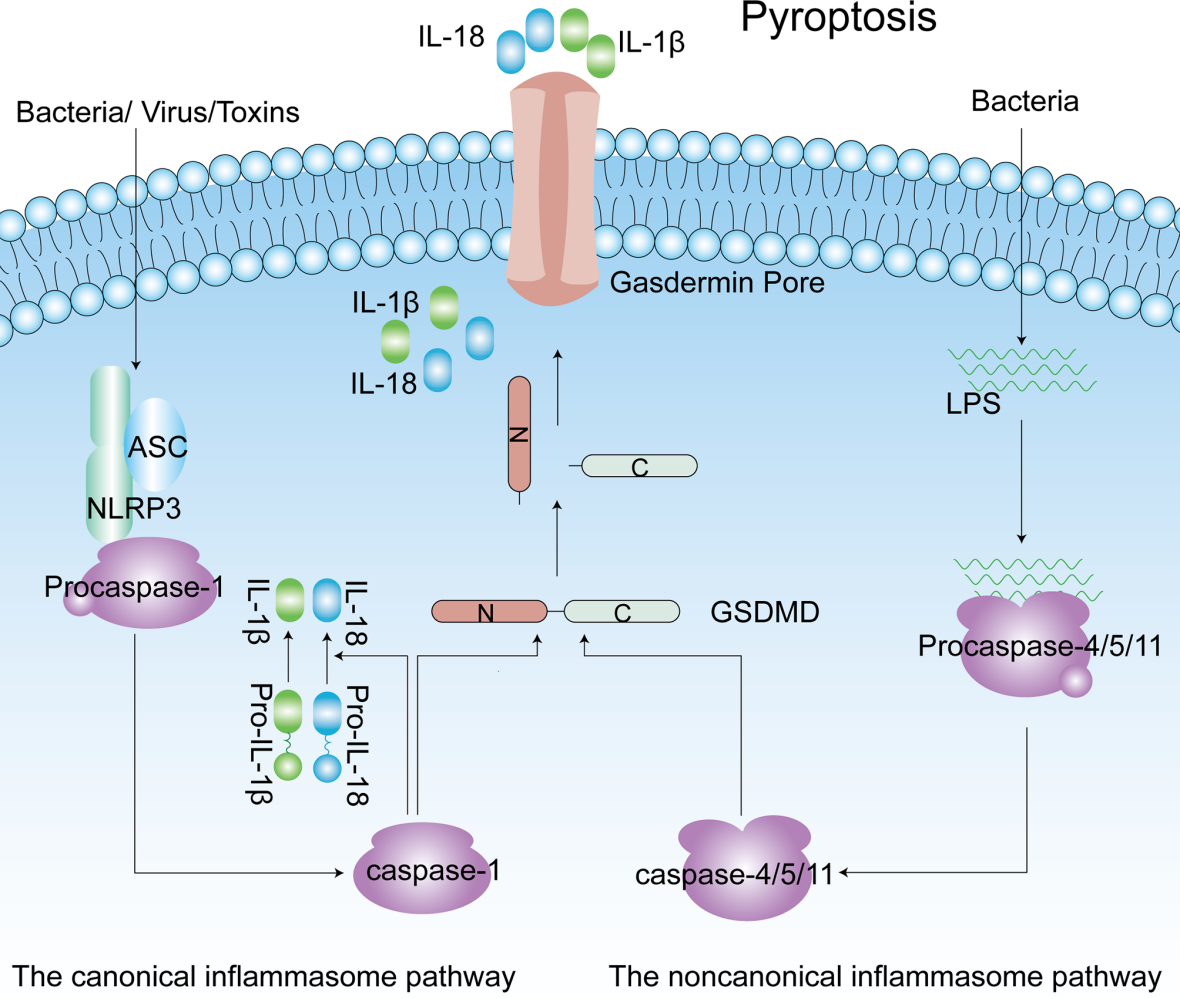
The canonical inflammasome pathway (Caspase-1-dependent) and noncanonical inflammasome pathway (Caspase4/5/11-dependent) of pyroptosis. Caspase-1 is activated by NLRP3 inflammasomes, and caspase4/5/11 are activated by direct interaction with LPS. Active caspase-1 and caspase-4/5/11 cleave the GSDMD to produce the C-terminus and N-terminus. Released gasdermin-N domains form an approximately 12–14 nm inner diameter pore on the plasma membrane. IL-1β and IL-18 are matured by active capase-1 and released from the gasdermin pore. ASC, apoptotic speck-like protein containing a caspase recruitment domain; IL-1β, interleukin-1β; LPS, lipopolysaccharide; GSDMD, gasdermin D; NLRP3, NLR family pyrin domain-containing 3.

Pyroptosis is a form of programmed cell death that has been identified in the past decade. Pyroptosis is crucial for innate immune defense, and it occurs in both macrophages and other cells (67). In contrast to apoptosis, pyroptosis is associated with inflammation. Some characteristics of pyroptosis are cell swelling, and IL-1β and IL-18 release from gasdermin pores in membranes (68). In pyroptosis, the caspase-1-dependent pathway is called the canonical inflammasome pathway, and the caspase-4/5/11-dependent pathway is described as the noncanonical pathway (69, 70). Diverse infections and immune challenges activate caspase-1 in cells through different inflammasomes, including NLRP3, NLRP1, apoptotic speck-like protein containing a caspase recruitment domain (ASC), NOD-like receptor family, caspase recruitment domain (CARD) containing 4 (NLRC4) and absent in melanoma 2 (AIM2) (71). Unlike caspase-1, intracellular lipopolysaccharide (LPS) directly interacts with caspase-4/5/11, and then the latter is activated (72–74). The common result of caspase-1/4/5/11 activation is that gasdermin D (GSDMD) becomes a pyroptotic effector of these caspases. Subsequently, GSDMD is cleaved to produce two parts: the N-terminus and the C-terminus. The N-terminus of GSDMD induces pore formation in the membrane, and these pores become the channels through which IL-1β and IL-18 are released, ultimately leading to cell death (**Figure 3**) (67, 75). More interestingly, caspase-11-mediated maturation of GSDMD triggers caspase-1 activation, accompanied by the secretion of IL-1β (76). In addition, GSDMD is not the only substrate of pyroptosis. In some cases, activation of gasdermin E (GSDME) by caspase-3 has also been shown to induce pyroptotic cell death (77, 78). In the innate immune system, pyroptosis exerts a dual effect. It protects the body from pathogen infection and endogenous threats but causes harmful inflammation in the case of excessive activation (68). As more in-depth research on pyroptosis has been conducted, pyroptosis has been found to be associated with the occurrence and development of many common diseases, including obesity, type 2 diabetes mellitus (T2DM) and complications of diabetes (79–81).

### 3.1 Pyroptosis in Obesity and T2DM

As living standards improve, the number of people with metabolic diseases is increasing annually (82). Obesity and DM are common metabolic diseases worldwide. As mentioned above, NLRP3 inflammasomes activate caspase-1 to trigger pyroptosis. Eventually, IL-1β and IL-18 are secreted by pyroptotic cells. Many studies have found that NLRP3 inflammasomes correlated with pyroptosis participate in the pathogenic mechanism of some metabolic diseases, including obesity and type 2 diabetes mellitus (83).

Obesity is a metabolic disorder with multifactorial pathogenesis. In addition, obesity is also a risk factor for T2DM. Macrophages of monocyte origin infiltrate tissues as one of the pathological changes of obesity. Monocytes in the peripheral blood of obese patients exhibit high inflammatory caspase activity. In monocytes, saturated fatty acids activate caspase-4/5 to induce the production and release of IL-1β and IL-18, eventually leading to obesity-related inflammation (84). Moreover, higher expression of NLRP3 is detected in adipose tissue from obese individuals than in adipose tissue from metabolically healthy individuals (85). Of note, *NLRP3* knockout prevented mice fed a high-fat diet from becoming obese (86). Adipocytes not only store energy but also secrete adipokines to regulate metabolism (87). Adipocyte dysfunction (e.g., decreased levels of insulin-sensitive adipokines and increased levels of proinflammatory cytokines) are related to insulin resistance and T2DM (88). Pancreatic β-cell dysfunction and insulin resistance are the main characteristics of T2DM. Previous studies have suggested that the NLRP3 inflammasome is closely associated with the pathogenesis of T2DM (89). HG, free fatty acids and a high-fat diet promote the activation of the NLRP3 inflammasome in patients with T2DM (90). Activation of NLRP3 inflammasomes induces excessive secretion of IL-1β and IL-18. Increased IL-1β levels cause the dysregulation of blood sugar levels by impairing pancreatic β-cells and inducing insulin resistance (83). The high expression of IL-1β receptors on pancreatic β-cells not only contributes to increase production of IL-1β, but also facilitates the spread of inflammatory signals through the NF-κB pathway, eventually leading to pancreatic β-cell dysfunction (91). IL-1β promotes insulin resistance by reducing the tyrosine phosphorylation and mRNA expression of insulin receptor substrate-1, and inducing the expression of tumor necrosis factor α (92). Additionally, IL-1β and IL-18 decrease the insulin sensitivity of target organs by inducing lymphocytes to accumulate in adipose tissue (93). In conclusion, NLRP3 inflammasomes are critical for the development and progression of obesity and T2DM, especially IL-1β, a product of NLRP3 inflammasome activation. Importantly, pyroptosis is associated with the occurrence and development of DM but also with the development of its complications, such as diabetic cardiomyopathy (94), diabetic nephropathy (95) and DR (96).

### 3.2 Pyroptosis in Diabetic Complications

#### 3.2.1 Diabetic Retinopathy

Diabetic retinopathy is prevalent among people with long-term DM and remains the critical cause of visual impairment in working-aged people. Loukovaara et al. used immunohistochemistry and observed that the levels of caspase-1 and IL-18 were significantly increased in DR patients’ vitreous (97). Simultaneously, they found that NLRP3 inflammasome activation plays an important role in the pathogenesis of proliferative DR (97). Numerous studies have reported that the expression levels of inflammatory components including NLRP3, ASC, procaspase-1, IL-1β, and IL-18, were significantly upregulated in diabetic rat retinal tissues compared to control group (98, 99). In a recent study, the authors documented that HG promoted RPE cell pyroptosis, and methyltransferase-like protein 3 (METTL3) could reverse these changes by targeting the miR-25-3p/PTEN/Akt signaling pathway (100). In addition, researchers have observed that HG promotes NLRP3 inflammasome activation and pyroptosis in HG-induced human retinal microvascular endothelial cells (HRMECs) and human retinal pericytes (HRPs) (101, 102). Several reports have suggested that P2X7 purinergic receptor (P2X7R) promotes DR pathogenesis (103, 104). P2X7R activates the NLRP3 inflammasome and promotes the release of the proinflammatory cytokine IL-1β in retinal pericytes treated by HG (105, 106). JNJ47965567, a P2X7R antagonism, can revert the damage caused by HG in cultured pericytes (104). Similarly, previous researchers have documented that H3 relaxin inhibits AGE-induced HRMEC pyroptosis by attenuating the P2X7R/NLRP3 pathway (107). In addition, researchers found that fenofibrate and sulforaphane provide significant protection against DR by attenuating NLRP3 inflammasome activation and activating the antioxidative Nrf2 pathway (108, 109). A recent study found that vitamin D3 exerts protective effects against DR by inhibiting ROS/TXNIP/NLRP3 inflammasome pathway activation (110). Similarly, a recent study demonstrated that vitamin D3 protects RGCs by reducing inflammatory cytokines and increasing the expression of neuroprotective factors in glaucomatous mice (111). Based on the above studies, pyroptosis may play a crucial role in the changes in retinal cells in the DR environment.

#### 3.2.2 Other Complications

Diabetic cardiomyopathy (DCM) is a crucial complication of DM and can result in heart failure (112). Growing research suggests that pyroptosis may be involved in the pathogenesis of DCM (113). Myocardial ultrastructure studies demonstrated that dying cells exhibited swollen fibril and mitochondria in the myocardium of diabetic rats, similar to the phenotypic features of pyroptosis (114, 115). Protein expression levels of the NLRP3 inflammasome, caspase-1, IL-1β and GSDMD were remarkably elevated in diabetic mouse cardiac tissue (94). In line with this, Ye et al. found that the mRNA levels of NLRP3, caspase-1 and IL-1β were considerably higher in the T2DM mice hearts than in control mouse hearts (116). In addition, a recent study showed that silencing long non-coding RNA (lncRNA) Kcnq1ot1 ameliorated pyroptosis and fibrosis in myocardial tissues of diabetic mice and was related to the Kcnq1ot1/miR-214-3p/caspase-1/TGF-β1 signaling pathway (94). Hyperglycemia induced cardiomyocyte pyroptosis in high-fat diet-induced T2DM mice *via* the AMPK-TXNIP pathway (117). Furthermore, other studies showed that AIM2 expression was significantly elevated in the heart tissue of diabetic rats compared to the control group (118). AIM2 is involved in HG-induced DCM cell death and fibrosis through the GSDMD pathway (118). These studies illustrate that pyroptosis may be an important contributor to the pathogenesis of DCM.

Diabetic nephropathy is a microvascular complication of DM and remains the major cause of chronic kidney disease throughout the world (119). Accumulating evidence demonstrates that pyroptosis plays a pivotal role in the progression of diabetic nephropathy (120). Recent studies have revealed that the protein levels of the NLRP3 inflammasome, GSDMD, caspase-1, and IL-1β in the kidney were significantly increased in diabetic rats and mouse models compared to the control group (95, 121). In addition, the expression levels of pyroptosis-associated proteins, such as caspase-11 or caspase-4, GSDMD, IL-1β and IL-18, in human and mouse podocytes cultured in HG are augmented (122). Caspase-4 or GSDMD knockdown considerably reversed these changes (122). Studies have confirmed that hyperglycemia promotes HK-2 cell pyroptosis (123). The lncRNA MALAT1 promotes hyperglycemia-induced HK-2 cell pyroptosis by inhibiting the expression of miR-23c, leading to the activation of the ELAVL1/NLRP3 pathway (123). Current studies have documented that the expression of lncRNA GAS5 in HG-stimulated HK-2 cells is repressed (124). In addition, GAS5 suppression significantly increased the expression of NLRP3, caspase1, IL-1β and GSDMD, and GAS5 overexpression reversed these changes (124). These notable findings indicate that pyroptosis may promote diabetic nephropathy pathogenesis.

## 4 Effect of Pyroptosis on the Retinal NVU in DR

### 4.1 Pyroptosis in Retinal Pericytes and Endothelial Cells

One of the earliest hallmarks of DR is microvascular changes, accompanied by the loss of pericytes, basement membrane thickening and the destruction of tight junctions between endothelial cells, together with hyperpermeability, capillary nonperfusion, microaneurysms, and the subsequent loss of endothelial cells (125, 126). Pericyte and endothelial cell death is a fatal blow to the retinal microvasculature. Multiple forms of cell death have been observed in diabetes-induced pericytes and endothelial cell death. Based on previous evidence, pericytes may die due to apoptosis and necrosis, and endothelial cells predominantly undergo apoptosis during the development of DR (127). However, some studies have found that pyroptosis might participate in the death of vascular cells in the retina and the pathological changes in DR.

Recently, HG was revealed to significantly induce the release of inflammatory cytokines and pore formation HRPs, resulting in pericyte lysis (102). Based on these findings, HG induces inflammation and pyroptosis in HRPs. Furthermore, HG induces retinal pericyte pyroptosis through the NLRP3-caspase-1 pathway (102). Coincidentally, HRPs undergo caspase-1-dependent pyroptosis after treatment with advanced glycation end product modified bovine serum albumin (AGE-BSA), which often appears in the diabetic environment (128). LncRNA myocardial infarction-associated transcript (MIAT) regulates caspase-1 expression by sponging miR-342–3p, ultimately resulting in the pyroptosis of HRPs treated with AGE-BSA (128). Notably, the authors used immunofluorescence staining and observed that AGE-BSA-induced HRPs exhibited phenotypic features of pyroptosis, including pyknosis, cell swelling, and hyperpermeability in the plasma membrane (128). Chen et al. documented that the protein expression of caspase-1, NLRP3, ASC, IL-1β, and IL-18 was significantly upregulated in retinal tissues of streptozotocin-induced diabetic rats (129). Furthermore, HG activated the NLRP3 inflammasome in HG-exposed HRMECs by the ROS-TXNIP pathway (129). Other studies have also shown that HRMECs undergo pyroptotic cell death under diabetic-like conditions (107). Platania et al. suggested that some miRNAs, such as miR-20a-5p, miR-20b and miR-106a-5p, are dysregulated in the retina and blood circulation of diabetic mice. These miRNAs can modulate the expression of DR-related factors, such as vascular endothelial growth factor (VEGF), participating in the progression of DR (130). Our group has reported that HRMECs cultured under HG conditions suffer from pyroptosis. Notably, miR-590-3p targets NLRP1 and inactivates the NOX4 signaling pathway to inhibit pyroptosis in HRMECs (101). In addition, prostaglandin E2 (an inflammatory mediator) participates in the activation of NLRP3 inflammasomes in HRMECs (131). Mcc950 selectively inhibits NLRP3 inflammasomes, thereby inhibiting human retinal endothelial cell (HREC) dysfunction under HG conditions (132).

The presence of gasdermin pores on the cell membrane is one of the characteristics of pyroptotic death. These pores destroy the osmotic potential, causing the cells to swell and eventually lyse (133). Diabetic environments such as HG and AGE-BSA promote retinal microvascular cell loss through pyroptosis (107). The loss of these two types of cells results in decreased pericyte-endothelial interactions and their miscommunication and contributes to microvascular instability. In addition, the loss of pericytes contributes to the formation of acellular capillaries, capillary occlusions, microaneurysms and hemorrhage (134). These significant pathological changes occur in DR. Vascular occlusion may lead to perfusion failure and retinal ischemic-hypoxic injury. The latter increases the expression of VEGF in glial cells and endothelial cells. Moreover, vascular occlusion may lead to retinal neuron dysfunction and even neuronal death. Furthermore, vascular cell death causes the destruction of the BRB (135), which increases vascular permeability and the possibility of inflammatory cells entering the retinal microenvironment. These processes are also the main features of DR (25). Alterations in BRB integrity lead to diabetic macular edema, eventually resulting in a severe visual impairment without timely intervention (136). Hyperglycemia-induced pyroptosis in the retinal microvasculature not only causes the death of pericytes and endothelial cells but also increases the number of inflammatory mediators, including IL-18 and IL-1β (102, 107). Researchers found increased levels of proinflammatory mediators in the serum or aqueous humor in patients with DR compared with normal controls (20). These inflammatory cytokines participate in triggering an even more excessive inflammatory reaction, promoting the development of DR (66, 137).

### 4.2 Pyroptosis in Retinal Neurons

Neurons in the retinal NVU are the major cells that transmit light signals and form vision. Extensive studies have shown the presence of retinal neuronal degeneration in the early stage of DR, even earlier than visible vasculopathy (138, 139). Two features of neurodegeneration in the retina are neuronal death and reactive gliosis (9). Many researchers have provided a description of neuronal apoptosis in DR. Pyroptosis, a form of programmed cell death involved in inflammation, can occur in retinal ganglion cells (140). DM causes hypoxia in retinal tissue and leads to an imbalance in retinal immune responses (141). Hypoxia-induced factor-1 (HIF-1) is continuously produced and degraded under hypoxic conditions. Then, HIF-1α functions as a transcription factor to activate the genes encoding the proangiogenic growth factors, IL-6 and IL-8 (141). Moreover, loss of pericytes potentially leads to acellular capillary formation, which is associated with vascular occlusion and leads to nonperfusion and retinal ischemic-hypoxic injury. Ischemia-hypoxia upregulates the expression of HIF-1 (25). Pyroptosis participates in retinal ischemic damage and promotes retinal ganglion cell death in acute glaucoma (142). The caspase-8-HIF-1α-NLRP12/NLRP3/NLRC4 pathway initiates neuroinflammation and pyroptosis (142). Moreover, pyroptosis is an alternative pathway through which photoreceptors degenerate after retinal detachment (143). Additionally, caspase-1/3/4/5 activities were found to be increased in a streptozotocin-induced diabetes mouse model (140). A previous study reported that NLRP3, ASC, and caspase-1 were specifically located in the GCL and the INL and ONL in the retinas of diabetic rats according to immunohistochemical results (99). Simultaneously, other authors have also documented that the expression levels of NLRP3, ASC, and caspase-1 were increased in retinal cells of diabetic rats (99).

### 4.3 Pyroptosis in Müller Cells

Müller cells and astrocytes of the retinal NVU participate in retinal structural support and maintain retinal homeostasis. Studies have found that approximately 15% of Müller cells die after 7 months in retinas of the diabetic mice (127). After exposure to HG, caspase-1 activity and IL-1β production in Müller cells increase and subsequently induce cell death (144). Furthermore, inhibition of the caspase-1/IL-1β pathway prevents the loss of Müller cells under diabetic conditions (127). The aforementioned evidence revealed that pyroptotic death may be responsible for Müller cell death under diabetic conditions. Consistently, HG-induced nuclear accumulation of GAPDH in Müller cells relies on activating the caspase-1/IL-1β pathway. More interestingly, the accumulation of GAPDH in the nucleus is associated with the induction of cell death (145). Due to their important locations and functions, Müller cell loss will lead to an incomplete retinal structure. For example, Müller cell loss promotes the destruction of the internal BRB integrity and increases the vascular permeability and the loss of neuroprotective effects, affecting both neurons and blood cells (35). Specific removal of Müller cells from the retina leads to retinoschisis, showing that Müller cells hold the neural layers together to protect the neural tissue from ripping apart (146). Previous studies have reported that selective removal of Müller cells results in photoreceptor apoptosis, BRB breakdown, and vascular telangiectasis (147). In addition, destruction of the cell-to-cell communication between Müller cells and retinal pericytes promotes pericyte death (148). The loss of Müller cells in DM is also related to the formation of aneurysms, a clinical feature of DR (35). Furthermore, Müller cells are the major source of IL-1β (149). Long-term production of IL-1β from Müller cells affects the viability of endothelial cells in a paracrine manner (150). Due to the high sensitivity of endothelial cells to IL-1β, endothelial cells die after responding to this inflammatory cytokine (150). In addition, the death of endothelial cells promotes the formation of acellular capillaries, which are the main hallmark of DR pathology (151).

### 4.4 Pyroptosis in Retinal Microglia

Microglia are specific innate immune cells of the retina that monitor the environment and remove metabolic waste. As more in-depth research on pyroptosis has been conducted, pyroptosis has also been identified in microglia. Microglial pyroptosis occurs in various disease states, such as after spinal cord injury (152), ischemic brain injury (153), retinal ischemia and reperfusion injury (154), and DR. Retinal ischemia and reperfusion injury (I/R) is the basis of multiple retinal diseases, including DR, glaucoma, and retinal artery occlusion (155). Accumulating studies have shown that I/R promotes retinal microglial pyroptotic death, which is associated with lncRNA H19 (154). In addition, S100A12 is closely related to the incidence and severity of DR. S100A12 represents a proinflammatory trigger for retinal microglial activation by activating the NLRP3 inflammasome in a diabetic environment (156). Moreover, HG was recently shown to induce retinal microglial pyroptosis through NLPR3 inflammasome signaling (157). HG upregulated the protein expression of NLPR3, caspase-1, GSDMD, and IL-1β in retinal microglia (157). In another study, HG-induced retinal cells produced more IL-1β, and the IL-1β induced microglial proliferation (158). Moreover, IL-1β is mainly produced by microglia under diabetic conditions (159). Overactive microglia produce various proinflammatory and cytotoxic factors, including IL-1β, TNF-α, and ROS, which lead to chronic inflammation and contribute to the destruction of hemostasis in the NVU, BRB breakdown and worsening of the pathology of DR (160).

### 4.5 Proinflammatory Mediators Produced From Pyroptosis in the NVU

Accumulating research suggests that IL-1β and IL-18 may partly come from the pyroptosis-mediated cell death of retinal cells in diabetic rats and mice (99). IL-1β and IL-18 are the two key cytokines that undergo maturation through cleavage by active caspase-1 and are released through pyroptosis. In addition, IL-1β is the most studied IL-1 family member in retinopathy, such as DR (161). These proinflammatory mediators lead to persistent low-grade inflammation, affecting the hemostasis of the retinal NVU. Under HG conditions, IL-1β induces pericyte apoptosis by activating NF-κB, thereby increasing vascular permeability (162). Additionally, IL-1β affects glial cells (microglia and macroglia) and ultimately results in neural changes (158). Moreover, the increased levels of IL-1β coincide with increasing retinal neovascularization (163). Coincidentally, IL-18 may promote retinal angiogenesis in active PDR together with VEGF or through VEGF (164). Accumulated studies indicate that glucocorticoids show anti-inflammatory efficacy by inhibiting key proinflammatory mediators such as IL-1β (165). Thus, glucocorticoids may have potential use in modulating pyroptosis. Collectively, IL-1β and IL-18 produced during pyroptosis participate in inflammation, promoting the progression of DR.

## 5 Conclusions

Pyroptosis is an emerging type of inflammatory cell death. Recent studies suggest that pyroptosis is involved in the pathogeneses of many diseases, such as obesity, T2DM and complications of diabetes. The retinal NVU is the functional basis of the retina. The proper function of every element of this retinal NVU is critical for normal retinal function. Impairment of the retinal NVU may result in abnormal physiological functions and even retinal disorders. Under diabetic conditions, most retinal NVU cells undergo pyroptotic cell death. Pyroptosis leads to cell death and promotes damaged cells to release various proinflammatory mediators, including IL-1β and IL-18. The accumulation of these proinflammatory factors promotes the formation of an inflammatory environment, further damaging the retinal cells and aggravating retinopathy. Inhibition of pyroptosis in retinal cells may be a treatment strategy for DR. The development of drugs targeting pyroptosis may provide benefits to the vast number of patients with DR. However, published research about the potential molecular mechanism and underlying role of pyroptosis-mediated cell death in retinal NVU cells is currently limited. Additional studies are necessary to investigate the fundamental role of pyroptosis in DR.

## Author Contributions

All authors contributed to the literature search writing and design of the manuscript. All authors have read and agreed to the published version of the manuscript.

## Funding

This work has been supported by the National Natural Science Foundation of China (81970811), Domestic Science and Technology Cooperation Project of Shanghai Municipal Science and Technology Commission (21015800700) and National Key R&D Program of China (2019YFC0840607).

## Conflict of Interest

The authors declare that the research was conducted in the absence of any commercial or financial relationships that could be construed as a potential conflict of interest.

## Publisher’s Note

All claims expressed in this article are solely those of the authors and do not necessarily represent those of their affiliated organizations, or those of the publisher, the editors and the reviewers. Any product that may be evaluated in this article, or claim that may be made by its manufacturer, is not guaranteed or endorsed by the publisher.
